# *Samplify*: a versatile tool for image‐based segmentation and annotation of seed abortion phenotypes

**DOI:** 10.1111/nph.70979

**Published:** 2026-02-15

**Authors:** Heinrich Bente, Ronja Lea Jennifer Müller, Andreas Donath, Dirk Walther, Claudia Köhler

**Affiliations:** ^1^ Department of Plant Reproductive Biology and Epigenetics Max Planck Institute of Molecular Plant Physiology Potsdam 14476 Germany

**Keywords:** Arabidopsis, image analysis, seed abortion, seed phenotyping, triploid block

## Abstract

- Automated seed phenotyping has wide applications in research and agriculture and relies on easy‐to‐use platforms and pipelines. Seed phenotyping in the model species *Arabidopsis thaliana* poses a significant challenge due to the large number of tiny seeds produced by individual plants, which are difficult to manually separate and count. Manual counting methods are time‐consuming and prone to user bias, particularly for subtle phenotypic changes.
- To address these limitations, we developed *Samplify*, a scalable, automated pipeline for seed segmentation and classification by integrating classical image processing techniques with Meta's Segment Anything Model.
- *Samplify* effectively segments *Arabidopsis* seeds, even in dense clusters where conventional methods fail. To demonstrate its versatility, we quantified the seed abortion occurring in interploidy crossings in Arabidopsis, often referred to as ‘triploid block’. *Samplify* includes a random forest classifier trained on a set of computed seed shape features that enable the categorization of seeds into normal, partially collapsed, and fully collapsed seeds, automating the manual classification process.
- The tool, designed as a command‐line application, significantly reduces manual annotation workload. Our validation across multiple datasets demonstrates high segmentation and classification reliability, making *Samplify* a valuable resource for the plant research community.

## Introduction

Seed development in flowering plants (angiosperms) is initiated by a process known as double fertilization, during which two haploid sperm cells from the pollen fertilize the two female gametes, the egg cell and the central cell, resulting in the formation of the embryo and endosperm, respectively (Dresselhaus *et al*., 2016). In angiosperms, the central cell is predominantly homodiploid, forming a triploid (3×) endosperm that inherits two copies of the maternal and one of the paternal genome (2 m:1p). This specific genome ratio in the endosperm is essential for the survival of the embryo in the majority of angiosperms. In *Arabidopsis thaliana*, as in most angiosperms, the endosperm initially develops as a coenocyte for a defined number of mitotic cycles, after which it starts to cellularize (Boisnard‐Lorig *et al*., 2001). Endosperm cellularization is critical and ensures proper embryo growth; cellularization failure will cause embryo arrest and seed abortion (Hehenberger *et al*., 2012). Changing the parental genome ratio in the endosperm affects endosperm cellularization and embryo development in F1 seeds. Pollinating a tetraploid maternal plant with a diploid pollen donor results in 3× embryos and 5× endosperms. The endosperm of those seeds cellularizes precociously, leading to smaller seeds that in Arabidopsis are nevertheless viable (Scott *et al*., 1998). The reciprocal cross of maternal diploid plants with pollen from a tetraploid plant results in 3× embryos and 4× endosperms. These seeds are characterized by delayed or failed endosperm cellularization, resulting in seed abortion. This phenomenon is often referred to as ‘triploid block’ (Marks, 1966; Köhler *et al*., 2010) and acts as a postzygotic hybridization barrier in a wide range of taxa (Coughlan, 2023; Bente & Köhler, 2024). Thus far, the genetic basis of the triploid block has been mainly investigated in Arabidopsis (Kradolfer *et al*., 2013; Martinez *et al*., 2018; Batista *et al*., 2019; Satyaki & Gehring, 2019).

Quantifying seed abortion typically involves manually photographing seed populations and categorizing seeds based on deviations in shape and color. Abnormal seeds are classified as either partially or fully collapsed: partially collapsed seeds appear misshapen but contain a developed embryo that often germinates, while fully collapsed seeds are dark brown and shriveled and mostly fail to germinate. However, this manual assessment is time‐consuming and inherently subjective, introducing variability and potential biases into the data.

To minimize observer bias, we developed an analysis pipeline based on automated image analysis, a critical step in quantifying seed developmental failure. Image analysis generally can be divided into two separate steps: segmentation, the partitioning of a given image into meaningful regions, and classification of the afore‐identified regions. While classification involves relatively simple tasks, such as size measurements, segmentation is the crucial prerequisite. Image segmentation itself can be typically classified into semantic segmentation, in which each pixel of an image gets a class label, instance segmentation, which identifies objects of the same class, whereas panoptic segmentation relies on a combination of the two (Kirillov *et al*., 2019). Segmenting images showing plant seeds is challenging because of high numbers and densely clustered seeds of different shapes that occasionally overlap. To circumvent this problem, different approaches were previously applied, ranging from manually dispersing seeds before imaging (Benjamaporn & Chomtip, 2017; Kurtulmuş, 2021), to the application of large particle flow cytometry for sorting single seeds (Morales *et al*., 2020), or using robotic approaches combining single seed sorting and imaging at the same time (Jahnke *et al*., 2016; Krzyszton *et al*., 2024; Klasen *et al*., 2025). Nonetheless, purely image‐based methods have become more prominent, as they offer affordable and standardized analysis without requiring specialized equipment. These methods have been mainly used for larger plant seeds, such as sugar beet (Wang *et al*., 2023) and soybean (Wei *et al*., 2023).

Alongside traditional image analysis tools, a range of specialized seed phenotyping pipelines have been developed, primarily targeting cereal crops and other large‐seeded species. Notable examples include ‘SmartGrain’ (Tanabata *et al*., 2012) and ‘GrainScan’ (Whan *et al*., 2014), which have been successfully applied to species, such as wheat, rice, millet, and *Brachypodium*. These tools are often optimized for high‐resolution input from flatbed scanners, which provide consistent lighting and minimize shadows, allowing for relatively straightforward segmentation methods, such as global or adaptive thresholding. However, such approaches typically assume well‐separated seeds and tend to remove or misclassify dense clusters of touching or overlapping seeds, which limits their applicability to species where seeds are small and more prone to aggregation during imaging. While handheld scanners have been explored as a more flexible imaging option, particularly for legumes like beans and peanuts (Huang *et al*., 2024), these devices often lack the resolution required to reliably capture the fine morphological features of smaller seeds. Additionally, many of these pipelines produce only basic shape or color descriptors (e.g. area, circularity, and Red, Green, Blue (RGB) values), placing the burden of biological interpretation and downstream statistical analysis on the user. More recently, the integration of artificial intelligence (AI) and deep learning has advanced seed image analysis by improving segmentation accuracy and enabling feature extraction from more complex or noisy images. These methods offer the potential to overcome challenges, such as overlapping seeds and nonuniform lighting conditions. However, most AI‐driven applications have so far been focused on larger‐seeded crops like rice, maize, or medicinal plants, such as *Scutellaria baicalensis* (Jinfeng *et al*., 2023; Keling *et al*., 2023) with relatively little attention given to small‐seeded species.

Thus, in the case of Arabidopsis seeds, which are usually small and come in high numbers, previous studies used manual quantification of phenotypes based on pictures, or dispersing seeds before imaging (Herridge *et al*., 2011; Ren *et al*., 2019; Merieux *et al*., 2021). To ease quantification of seed phenotypes, we developed *Samplify*, a command‐line tool designed for automated segmentation and classification of Arabidopsis seeds. The name reflects the integration of Meta's ‘Segment Anything Model’ (SAM), and the purpose of simplifying otherwise time‐consuming and user‐dependent seed classification. *Samplify*'s segmentation pipeline is designed in a hybrid segmentation approach, balancing computational efficiency and segmentation accuracy by combining classical image processing techniques with advanced transformer‐based models. The segmentation of regions where seeds are sparsely distributed and do not touch each other is achieved via traditional processing methods, namely Otsu thresholding (Otsu, 1979). However, in regions with dense clusters of seeds that touch or overlap, *Samplify* utilizes SAM (Kirillov *et al*., 2023), a transformer‐based model designed for general‐purpose segmentation. By leveraging SAM's capabilities, *Samplify* achieves high segmentation accuracy in challenging regions where conventional methods would typically require manual correction. Previous studies have demonstrated that implementing SAM‐based segmentation enhances performance, specifically in medical imaging (Wei *et al*., 2023; Ma *et al*., 2024; Junde *et al*., 2025; Li *et al*., 2025). Combining traditional image segmentation with SAM offers a scalable, efficient, and robust segmentation strategy. From each segmented entity, the tool collects multiple features, including shape, color, and texture that the user can access for downstream analysis. *Samplify* was specifically designed and tested for the quantification of seed abortion in *Arabidopsis thaliana*; thus, we included a random forest (RF) classifier that subsequently labels segmented seeds into normal, partially, or fully collapsed seeds. The underlying machine learning method builds many decision trees during training and combines their predictions to improve accuracy and reduce overfitting. The subsequent final class prediction of the RF is done by majority vote across all decision trees. Our provided RF model was trained on more than 13 000 seeds across 99 images that were all manually annotated, resulting in a robust RF model for seed abortion prediction. We tested *Samplify* using different independently generated datasets, demonstrating its high accuracy in segmentation and reliable seed abortion prediction. We believe that in combining automated segmentation together with a robust seed abortion RF classifier, *Samplify* reduced user bias inflicted inaccuracies as well as time‐consuming manual counting of such phenotypes, thus providing a valuable tool for the plant community. *Samplify*, including examples and instructions, are available on GitHub (https://github.com/Ronja‐Mueller/Samplify.git).

## Materials and Methods

### Plant material and growth condition

Columbia‐0 (Col‐0) and Landsberg *erecta* (L*er*‐0) *Arabidopsis thaliana* (L.) Heynh. seeds were obtained from the Nottingham Arabidopsis Stock Center and amplified for multiple generations in the laboratory. Autotetraploid 4× plants were generated via colchicine treatment of seedlings as described (Lafon‐Placette *et al*., 2017). Colchicine‐treated autotetraploid plants were amplified for at least three subsequent generations before use for crossings. The *phe1phe2* (Batista *et al*., 2019) in *osd1‐3* (Heyman *et al*., 2011) mutants as well as the *phe2* (SALK_105945; Batista *et al*., 2019) mutant were previously described.

All seeds were surface sterilized with 70% ethanol for 10 min, washed once in fresh 70% ethanol for 10 min, followed by a final wash in 100% ethanol for 5 min. Hereafter, ethanol was removed by pipetting and seeds were kept in opened tubes under sterile conditions until the remaining ethanol evaporated (*c*. 30 min). Surface‐sterilized seeds were plated on ½‐strength Murashige & Skoog medium plates containing 1% sucrose, 0.68% agar and stratified for 2 d in the dark at 4°C. Subsequently, seeds were germinated under long‐day conditions (16 h : 8 h, light : dark) at 21°C and 50 μE light intensity. Seedlings were transferred to soil after 7 to 10 d and grown in phytotrons under long‐day conditions (day 21°C, night 20°C, 70% humidity, 150 μE light intensity). Crosses were performed *c*. 3–4 wk after transfer, by emasculating individual flowers, and pollination 48 h later with appropriate pollen. Crosses were performed in at least three replicates, whereas each replicate is defined by independent maternal plants pollinated with independent paternal pollen donors. Each cross contained three to five siliques, resulting in *c*. 100–350 seeds. F1 seeds from single crosses were harvested in paper bags when siliques became brown, *c*. 3 wk after emasculation. F1 seeds were kept for 1 wk at room temperature before further experiments to ensure proper ripening. Seeds were released from the dry siliques by tapping against the paper bag for a few times. All content was poured on clean printer paper, and remaining siliques were removed with forceps. If necessary, seeds were stored in paper bags until imaging.

### Seedling establishment

For the quantification of seedling establishment, F1 seeds from inflorescences containing seeds from three to five siliques were evenly distributed on 9‐cm round Petri dishes containing 1% plant agar. Hereafter, seeds were stratified for 3 d at 4°C in the dark. Seedling establishment was imaged after 4 d of growth (16 h : 8 h, light : dark, 60 μE, 22°C). Manual counting of seedling establishment was done using ImageJ (v.1.54f; Schindelin *et al*., 2012) using ‘multi‐point tool’, whereas Counter 0 was used for germinating seeds and Counter 1 for nongerminating seeds. Differences in seedling establishment between genotypes were analyzed using a generalized linear model with a quasi‐binomial error distribution and a logit link function, with *post hoc* comparisons based on Tukey‐adjusted estimated marginal means (EMMs).

### Imaging of seed populations

Imaging of seed populations was done using a Keyence Digital Microscope VHX6000 using the ZS20 lens on either 20× Zoom or 50× Zoom if not stated otherwise. Seeds from single crosses were spread out *c*. 2 cm on clean white paper sheets, making sure that they did not lie on top of each other. Images were acquired in end point stitching mode, scanning an area of *c*. 40 mm × 30 mm to ensure white framing of the seed population. We applied full ring lighting and high exposure to ensure minimal shadows between the seeds. Scale bars were applied by the Keyence control software and were either 500 μm or 1 mm. Pictures were saved in JPEG format. Each experiment was imaged in one session and contained diploid selfed controls and triploid seeds to ensure the same lighting and edge length across all samples in one experiment. Manual counting for seed abortion was done using ImageJ (v.1.54f; Schindelin *et al*., 2012) using ‘Multi‐point tool’, with Counter 0 for counting normal seed, Counter 1 for the number of partially collapsed seeds, and Counter 2 for the number of fully collapsed seeds.

### *Samplify* – computational workflow for seed segmentation and classification

The *Samplify* pipeline integrates classical computer vision techniques with transformer‐based deep learning models. Images were first converted to grayscale and smoothed using Gaussian blurring (OpenCV 4.7.0; Bradski & Kaehler, 2008). Initial segmentation applied Otsu's thresholding (Otsu, 1979) and Canny edge detection (via OpenCV; Canny, 1986) to separate seeds in sparsely populated regions. For densely clustered seed regions, Meta AI's SAM (automatic mask generation mode; Kirillov *et al*., 2023) was used, implemented with PyTorch (v.2.0.1; Paszke *et al*., 2019) and run with GPU acceleration (CUDA 11.8; Nickolls *et al*., 2008). SAM parameters were manually optimized for seed segmentation (e.g. *points_per_side = 49* and *pred_iou_thresh = 0.86*).

Feature extraction for each segmented seed was performed using a parallelized approach (via Python's *concurrent.futures.ThreadPoolExecutor*) and included 38 descriptors: 21 color metrics, 9 shape descriptors (e.g. eccentricity, solidity, and overlap ratio), 5 Haralick texture features (via *skimage.feature.graycomatrix*), and 3 positional features (Supporting Information Table S1).

A RF classifier (Scikit‐learn 1.2.2; Pedregosa *et al*., 2011) trained on 13 395 manually labeled seeds from the *TripBlockDefault_RF* dataset was used to classify each seed into one of three categories: normal, partially collapsed, or fully collapsed. RF parameters were optimized using grid search, with a test size of 30%, stratified labels, and random state set to 42. The final RF classifier used 100 estimators, maximum depth of 7, minimum samples split of 5, bootstrap enabled, and out‐of‐bag scoring. Output included color‐coded annotated images, feature information for each segmented seed, and Excel summary files reporting class counts per image. The pipeline was optimized for high‐throughput processing of up to 300 seeds per image and achieved *c*. 60% reduction in execution time via batch‐wise GPU execution and feature parallelization. An overview of the *Samplify* workflow is presented in Fig. S1. *Samplify* was tested on two independent systems utilizing either an Intel(R) Xeon(R) CPU E5‐2640 v4 @ 2.40 GHz with 20 cores, 64 GB RAM, and a NVIDIA GeForce RTX 4070 GPU, or an Intel(R) Xeon(R) W‐2133 CPU @ 3.60 GHz with 12 cores, 32 GB RAM, and a NVIDIA Quadro RTX 4000 GPU. Additionally, we tested *Samplify* on a personal computer running (Intel(R) Core(TM) i5‐9400F CPU @ 2.90 GHz; NVIDIA GeForce RTX 4070 SUPER) Windows11 utilizing the Windows Subsystem for Linux (WSL, v.2.6.2.0) with Ubuntu 24.04 using the provided instructions. As *Samplify* occasionally recognized foreign objects, prediction images were manually checked for misannotations. Due to the low number of wrongly annotated objects, we did not apply corrections for full transparency.

To evaluate differences in seed abortion between genotypes, we used a multinomial logistic regression model (R package nnet; Venables & Ripley, 2013, Yee, 2015) fitted to the counts of seeds in the categories normal seeds, partially collapsed seeds and fully collapsed seeds. To account for extra‐multinomial variation, a dispersion factor was estimated from a Pearson χ^2^ statistic and used to scale the model covariance matrix (quasi‐multinomial approach). *Post hoc* comparisons among genotypes were performed within each phenotype category using EMMs and Tukey adjustment of the emmeans R package (Lenth, 2016). If applicable, pairwise comparisons between genotypes were conducted, and significance groupings were determined using compact letter displays from the multcomp R package (Hothorn *et al*., 2008). Statistical differences in size and color parameters were calculated via one‐way ANOVA and *post hoc* Tukey test. All analyses were performed in R (v.4.5.0; R Core Team, 2020), data were processed using the tidyverse R package (Wickham *et al*., 2019) and plotted via ggplot2 (Wickham, 2009). The multcompview package (Graves *et al*., 2019) was used for statistical group assignment. All *P*‐values and statistical groups are indicated. Raw values and full test statistics can be found in Tables S2–S18.

## Results

### *Samplify*, a training and annotation workflow for seed abortion

**Fig. 1:**
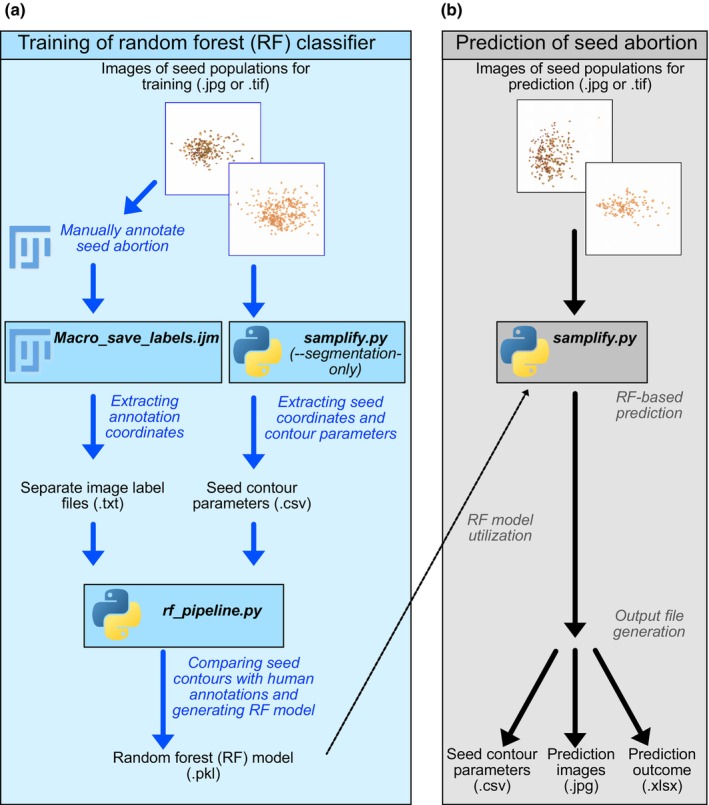
Workflow for *Samplify*‐based phenotyping of seed abortion. In order to use *Samplify*, users have to provide a directory with images in .jpg or .tif format for either training a random forest (RF) model or for predicting seed phenotypes. (a) For the generation of a RF model, each picture had to be separately counted using ImageJ multipoint tool as described and the label information has to be extracted using the *Macro_save_labels.ijm* macro of ImageJ. In the same directory, users can *run samplify.py ‐‐segmentation‐only* to generate seed contour parameters. The Image label files together with the seed contour parameter file is used to run the python script *rf_pipeline.py* to generate and test a RF model (.pkl) including testing parameters of the same. (b) By using a prior created RF model, users can start predicting seed abortion phenotypes on a set of pictures. By default, *samplify.py* performs a segmentation that outputs a seedparameter.csv file and a prediction outcome in .xlsx format. Moreover, for each input picture, an annotated prediction picture is created in a subdirectory to allow fast validation. Blue arrows and annotations indicate the training path; black arrows and annotations indicate the segmentation and prediction path. Blue and grey boxes indicate different scripts for each of the tasks utilized; plain text describes different output files, with the file ending in brackets; italicized text represents additional information about the process.

*Samplify* is a purely image‐based seed phenotyping platform, requiring users to provide only preannotated pictures for training and seed abortion prediction (Fig. 1a). The *Samplify* pipeline consists of two steps, namely a ‘training’ and ‘prediction’ step, both necessary to achieve automated seed phenotyping. As *Samplify* relies on a machine learning approach, users first need to use their pictures to train a RF model before being able to start the prediction. The training step consists of multiple smaller steps as indicated by the blue arrows in Fig. 1(a).

Pictures used for training need to be manually annotated, which can easily be done using the Multipoint tool in ImageJ (Schindelin *et al*., 2012). For annotating pictures for the triploid block, we counted each picture of the training set using the Point tool, assigning ‘Counter 0’ for ‘normal seeds’, ‘Counter 1’ for ‘partially collapsed seeds’ and ‘Counter 2’ for ‘fully collapsed seeds’. To extract the label information of each picture, we used the ImageJ Macro *Macro_save_labels.ijm*, which needs to be downloaded and installed in the ImageJ software. The Macro accesses the directory with all annotated TIFF files and creates a TXT‐directory containing for each annotated TIFF file a separate image label file (.txt) with coordinates and categories for a given picture. As we opted to train the RF model only on proper segmented pictures, we added a segmentation‐only option in *Samplify*'s main program. Running *samplify.py ‐‐segmentation‐only* on a directory with training images (either raw JPGs or annotated TIFFs) results in *seed_parameters.csv* containing a table with all segmentation information (Table S1) from all images in the input directory.

In the segmentation process, *Samplify* will take each single picture and apply a hybrid segmentation model, trying to identify clusters of seeds and applying Metas ‘Segment Anything’ (SAM) image analysis to identify single seeds. Only for seeds not appearing in a cluster as they are too dispersed, *Samplify* utilizes traditional thresholding techniques to achieve segmentation of all individuals in one picture.

In the final training step, the *rf_pipline.py* file uses the *seed_parameters.csv* file from the *Samplify* segmentation and the TXT‐directory of the Separate image label files to create a RF model (.pkl) and testing statistics of that model. The whole training workflow is depicted by blue arrows in Fig. 1(a). After users have approved the results of the test model, it can be used for different predictions. This only requires the raw pictures, which should ideally be taken using the same settings as done for the training dataset. Users can run *samplify.py* in a directory containing the pictures to be analyzed. *Samplify* will automatically use the default RF model *TripBlockDefault_RF.pkl* as long as no other model is specified. As output, *Samplify* will generate a subdirectory called ‘out’ containing the *predicted_images* directory with all *Samplify*‐annotated images, as well as two files, a *seed_parameter.csv* file with all picture segmentation and image information. It will furthermore generate a *seed_summary.xlsx* file, which contains the number of annotated total seeds and seeds per phenotype category (‘normal’, ‘partially’, and ‘fully collapsed’), as well the relative values in percentage per picture. Moreover, the *seed_summary.xlsx* file contains further information on processing time and prediction confidence. An overview of the prediction process is depicted in Fig. 1(b).

### Training data of *Samplify*

**Fig. 2:**
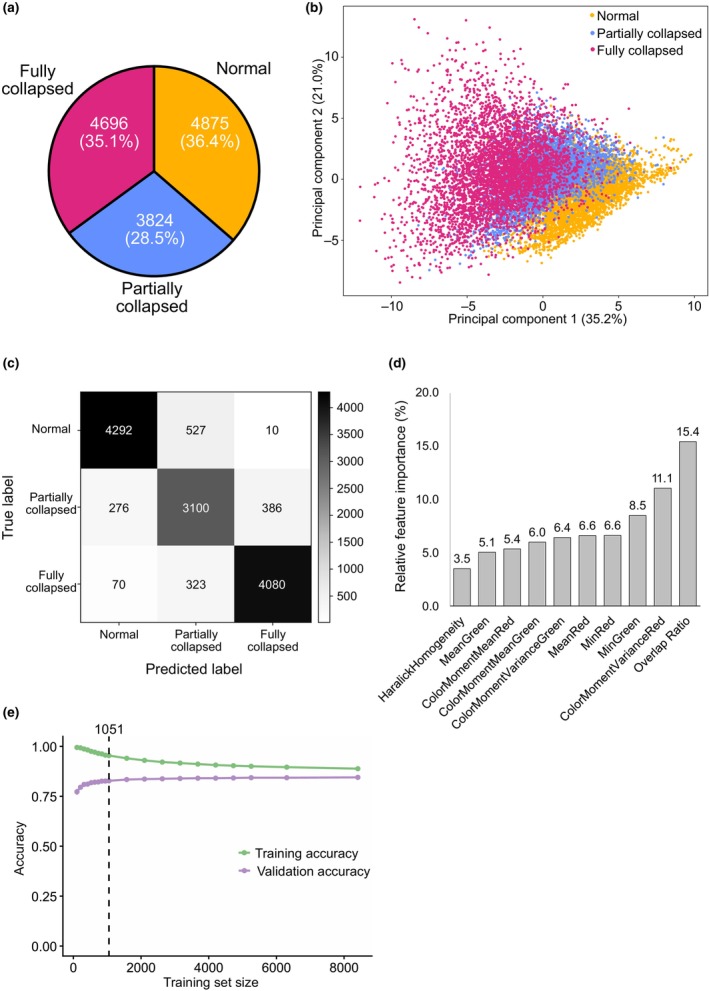
Default training dataset for *Samplify*. (a) PieChart depicting the total and relative amount of manually annotated seed data from 99 pictures containing a total of 13 395 seeds, which contain 36.4% normal, 28.5% partially collapsed, and 35.1% fully collapsed seeds. (b) Principal component analysis (PCA) across different manually annotated seed abortion categories in the training dataset. (c) Confusion Matrix of 13 064 seeds with overlapping label and segmentation info, illustrating the overlap of manually labeled and predicted seeds by *Samplify*. (d) Relative feature importance of the top 10 parameters used in the random forest (RF) classifier for the default model. (e) Prediction accuracy using randomly down‐sampled trainings sets. Green denotes prediction accuracy of seeds from the training set itself (*n* = 10 509 seeds). Pink denotes prediction accuracy of seeds manually annotated seeds independent from the training set used (*n* = 2628). The mean of 30 iterations from down‐sampled training sets is shown. Dashed line marks the point in of training set where the accuracy difference < 0.1% to the next bigger trainings set. A list including the size of all training sets can be found in Supporting Information Table S2. A full list of feature importance can be found in Fig. S2.

As *Samplify* was specifically developed for the segmentation and subsequent quantification of seed abortion, we trained an RF model on a comprehensive dataset consisting of 99 pictures, obtained from multiple triploid block experiments, in total containing 13 395 single seeds. The distribution across the different seed abortion categories showed that 36.4% of these seeds were manually labeled as normal, 28.5% as partially collapsed, and 35.1% as fully collapsed seeds (Fig. 2a). A principal component analysis (PCA) across the different manually annotated seed abortion categories showed that 35.2% and 21% of variation can be dissolved by PC1 and PC2, respectively (Fig. 2b). Furthermore, the PCA revealed that partially collapsed seeds were not clearly separated from viable and fully collapsed seeds, reflecting that these categories are sometimes not clearly distinguishable (Fig. 2b). Training the RF classifier on that dataset resulted in an accuracy of 87.8% across 13 064 single individuals that could be segmented properly and assigned to manually annotated labels. There was only a small percentage of seeds where predicted labels deviated from the actual labels, especially for partially collapsed seeds (Fig. 2c). Next, we tested the relative feature importance after training the RF classifier. Plotting the top 10 most important features to distinguish different seed abortion categories revealed that the overlap ratio, measuring the intersection‐over‐union of the seed shape to an ellipse fitted to its contour, was the top contributor with 15.4%, followed by red and green color parameters. Thus, the RF uses the same features for classification as subjectively assessed by human annotators (Figs 2d, S2). At last, we tested the required size of the training set to achieve accurate prediction. We split our training dataset into two parts, a ‘training pool’ with 10 509 seeds and a ‘validation pool’ with 2628 seeds. Next, we generated 20 differently sized random training sets from the training pool ranging from 105 to 8407 randomly selected seeds per set. The random set generation was done in 30 iterations, resulting in a total of 600 training sets. We tested seed predictions done with all 600 training sets and against all seeds from the training pool and against the independent validation pool (Fig. 2e). Whereas accuracy still varies in low training set sizes, a training set size of 1051 seeds was sufficient to reach a plateau, at which accuracy changes by less than 0.1% compared with the next larger set (Table S2). In conclusion, our trained RF model, based on 13 064 manually labeled seeds, demonstrates high accuracy in predicting normal and fully collapsed seeds. However, partially collapsed seeds are sometimes incorrectly annotated due to their shared features with both normal and collapsed seeds.

### Reliable prediction of seed abortion in interploidy crosses using *Samplify*

**Fig. 3:**
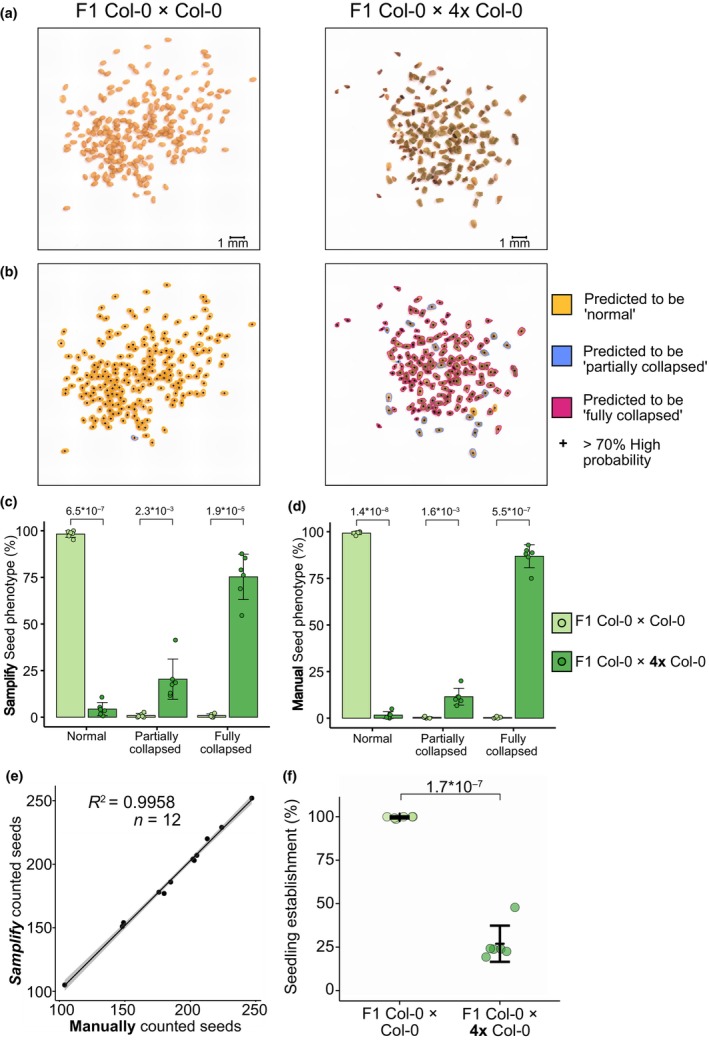
*Samplify* faithfully segments, annotates, and predicts seed abortion in control crosses. (a) Input pictures of F1 Col‐0 × Col‐0 (left side) and F1 Col‐0 × 4× Col‐0 (right side) seed populations and its annotation by *Samplify* (b). Scale bar = 1 mm. Relative seed phenotypes from F1 Col‐0 × Col‐0 and F1 Col‐0 × 4× Col‐0 annotated by *Samplify* (c) and manually annotated (d). Statistical comparison was done per category by applying quasi‐multinomial estimated marginal means (EMMs) with Tukey adjustment; *P*‐values of comparisons are plotted. Error bars represent SD. (e) Scatterplot of *Samplify* segmentation against the total number of manually counted seeds per picture. (f) Seedling establishment at 4 d of growth in F1 Col‐0 × Col‐0 and F1 Col‐0 × 4× Col‐0. Statistical comparison was done by applying quasi‐binomial EMMs with Tukey adjustment, *P*‐value as indicated. A full list of test statistics and values used can be in Supporting Information Tables S3–S6.

After having successfully established training and validation with the training dataset, we tested *Samplify*'s segmentation and prediction with independent triploid block data. For this test, we included pictures of F1 seeds obtained by crossing Col‐0 × Col‐0, resulting in normal, light brown, plumb seeds referred to as ‘diploid seeds’. Moreover, we included the same number of pictures from F1 seeds of Col‐0 × 4× Col‐0, a cross resulting in a high seed abortion rate, referred to as ‘triploid block’ (Scott *et al*., 1998). These collapsed seeds have a variable size and shape and are darker in color, specified as ‘triploid seeds’ (Fig. 3a). The majority of seeds from F1 Col‐0 × Col‐0 were predicted by *Samplify* as normal seeds, whereas F1 Col‐0 × 4× Col‐0 were predominantly predicted as partially or fully collapsed seeds (Fig. 3b). To obtain quantitative data, we applied *Samplify* using our default RF model on six replicates each for F1 Col‐0 × Col‐0 seeds and F1 Col‐0 × 4× Col‐0 and plotted the relative amount of normal, partially collapsed and fully collapsed seeds (Fig. 3c). As expected, diploid seeds were predicted to be predominantly (98.24%) normal, in contrast to only 4.3% of triploid seeds. Triploid seeds were predicted to be either partially collapsed (20.4%) or fully collapsed (75.3%), while these categories were rarely predicted for diploid seeds (< 1%, Fig. 3c; Table S3). These values are well in line with manual classification of seed abortion based on the same pictures, resulting on average in 99.3% normal, 0.4% partially and 0.3% fully collapsed diploid seeds, compared with 1.7% normal, 11.5% partially and 86.8% fully collapsed triploid seeds (Fig. 3d; Table S3).

To evaluate *Samplify*'s segmentation capability, thus recognizing single seeds in given pictures of seed populations, we compared the total number of manually counted seeds from all pictures *vs* the number of seeds segmented by *Samplify*. Single seeds counted manually and detected by *Samplify* nearly perfectly aligned, resulting in an *R* ^2^ value of 0.9958 across 12 independent seed populations (Fig. 3e). Manual counting resulted in 2236 seeds, whereas *Samplify* identified 2266 seeds, a difference of only 30 seeds and less than 2% variation (Table S5).

Finally, we tested whether seeds predicted as normal or partially collapsed can germinate, thus correlating the prediction of seed viability with the potential to germinate. Similar to the high proportion of normal diploid seeds predicted as well as manually counted, these seeds had a high germinability of 99.6% over all replicates. By contrast, triploid seeds had, on average, a germination rate of 26.9%, corresponding well to the predicted number of 20.4% partially collapsed seeds (Fig. 3f; Table S6), which were previously shown to largely keep their potential to germinate (Hehenberger *et al*., 2012; Batista *et al*., 2019). Indeed, the prediction of partially collapsed seeds based on *Samplify* comes closer to the actual number of germinating seeds than the number based on manual counting (11.5%), demonstrating the high prediction value of *Samplify*. Taken together, these results demonstrate that the automated segmentation and annotation of seed abortion is highly reliable and aligns and even outperforms traditional methods of manual counting, thus providing a fast and less biased way of quantifying seed abortion.

### *Samplify* generates reproducible results

**Fig. 4:**
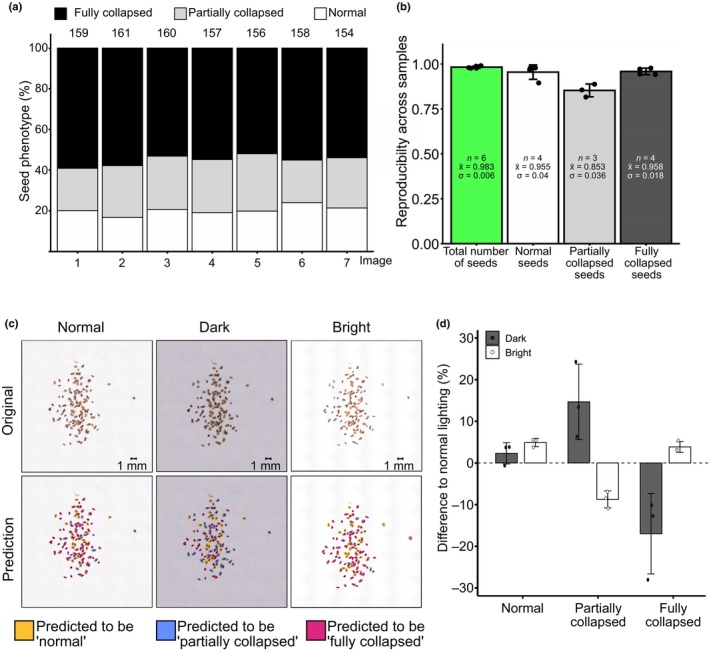
*Samplify* predictions are reproducible across different iterations. (a) *Samplify* quantified seed abortion on seven pictures of one seed population. Each picture was taken with the same setting and seeds were randomized in between acquisitions. Numbers on top represent the total amount of seeds detected by *Samplify*. (b) Reproducibly across different seed populations per scored category. Reproducibility was calculated by 1‐coefficient of variation (CV). Whereby CV is the SD (σ) divided by the mean (x̄) of each seed population in seven different pictures. To account for low numbers, reproducibility was plotted only for those populations that showed an average count > 10 in each category; *n* represents the number of populations matching this criterion. A full table of raw counts and reproducibility calculations can be found in Supporting Information Tables S7–S8. (c) Original pictures (upper panel) and *Samplify* prediction pictures (lower panel) of the same seed population at normal lighting conditions, under‐exposed settings (‘Dark’), and over‐exposed setting (‘Bright’). (d) Change in relative seed phenotype predictions at different lightning conditions as shown in (c). Scale bar = 1 mm. Three seed populations were imaged at three different light conditions and the relative change in seed phenotype per category is shown. Error bars represent SD. Relative values are denoted in Table S9.

To further test the prediction confidence of *Samplify* across different biological samples, we evaluated its reproducibility by comparing predictions of seed abortion in the same population using different images. We selected a seed population with normal, partially, and fully collapsed seeds and imaged these seeds using the same settings; however, after each image, the seeds were newly dispersed for the next image, resulting in multiple pictures of the same seed population. The predicted number of seeds as well as predicted categories was highly similar among the replicates, revealing low technical variation due to different seed positions (Fig. 4a; Table S7). To further test the technical variation of *Samplify*'s seed prediction, we measured the per‐category reproducibility across six random seed populations, each imaged in seven iterations. Reproducibility was calculated as 1‐coefficient of variation (CV), in which CV is the SD divided by the mean, SD/mean. Reproducibility was assessed for total seed number as well as for each seed phenotype category independently. To account for low numbers, reproducibility was calculated only for those populations that showed an average count > 10 in each category. This resulted in an average reproducibility of 0.983 for total seeds (*n* = 6), 0.955 (*n* = 4) for normal seeds, 0.853 (*n* = 3) for partially collapsed seeds, and 0.958 (*n* = 4) for fully collapsed seeds (Fig. 4b; Table S8). Together, this analysis shows high reproducibility for the prediction of total seed number, high reproducibility for normal and fully collapsed seeds, and good reproducibility for partially collapsed seeds. The fact that partially collapsed seeds show less reproducibility compared with the other categories is a consequence of their intermediate phenotype, sharing characteristics of normal and collapsed seeds.

We further tested whether reproducibility would be affected by imaging conditions, in particular, different light exposure and resolution. We tested *Samplify*'s seed phenotype prediction using different lighting conditions, by comparing our normal lighting condition with under‐exposed images referred to as ‘dark’ and over‐exposed conditions called ‘bright’ (Fig. 4c). *Samplify* does a background normalization; thus, all images were adjusted, resulting in perfect white backgrounds in the prediction images (Fig. 4c, lower panel). The fraction of normal seeds was the least affected category, with on average 2.3% more normal seeds predicted in dark conditions and 4.9% more in bright conditions. The prediction of partially collapsed seeds was more strongly affected by lighting conditions, with an average increase of 14.7% in dark conditions and a decrease of 8.7% in bright conditions. This substantial change in partially collapsed seed prediction was accompanied by a corresponding reduction in the annotation of fully collapsed seeds, which decreased by 17% in dark conditions and increased by 3.9% in bright lighting compared with normal light (Fig. 4d; Table S9). We also tested different image resolutions (ranging from *c*. 19 to 130 megapixel) on the same seed populations, which resulted in comparable prediction outcomes (Fig. S3; Table S10). These findings emphasize the importance of lighting in automated seed phenotyping using *Samplify*, and we recommend that users carefully control exposure conditions or incorporate suitable controls, such as well‐characterized seed populations with predefined abortion ratios. To achieve optimal results, a combination of both approaches is advisable.

### *Samplify* predicts seed abortion in triploid block suppressor mutants and different accessions

**Fig. 5:**
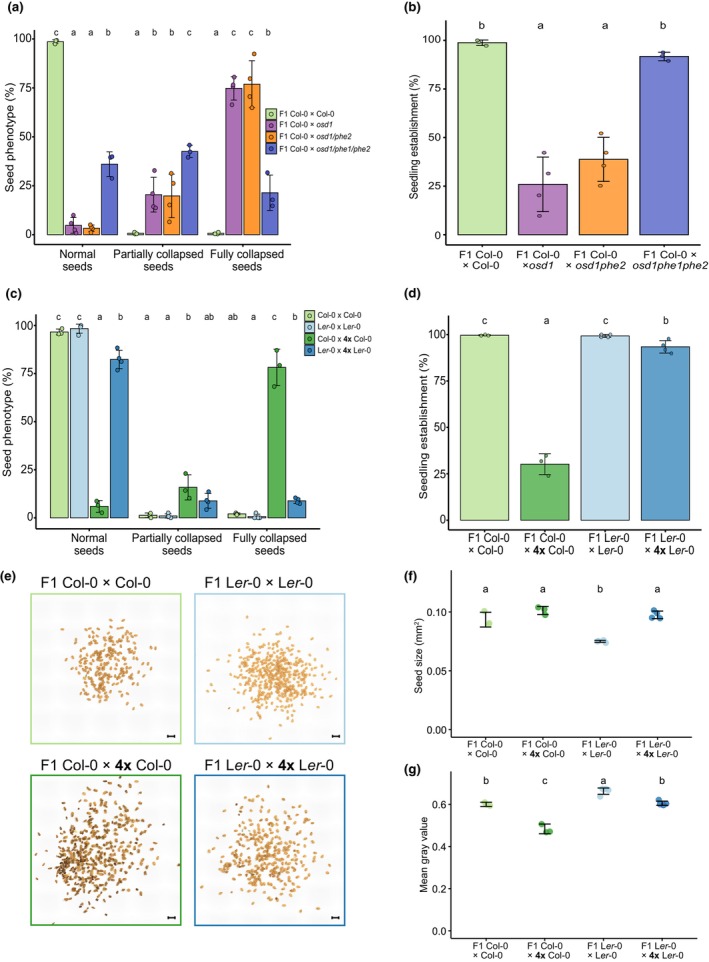
*Samplify* can identify triploid block suppressor mutants and accession‐specific differences. (a) *Samplify* predicted seed abortion phenotypes in seed populations from different genetic crosses, including relative seedling establishment at 4 d of growth (b). (c) Relative seed abortion phenotype as predicted by *Samplify* in F1 Col‐0 × Col‐0, F1 Col‐0 × 4× Col‐0, F1 L*er*‐0 × L*er*‐0, and F1 L*er*‐0 × 4× L*er*‐0, and seedling establishment at 4 d of those (d). Statistical comparison for seed phenotypes was done per category by applying quasi‐multinomial estimated marginal means with Tukey adjustment, statistical groups are plotted above (*P* < 0.05), for seedling establishment a quasi‐binomial approach was used. (e) Representative pictures of Col‐0 × Col‐0, F1 Col‐0 × 4× Col‐0, F1 L*er*‐0 × L*er*‐0, and F1 L*er*‐0 × 4× L*er*‐0. Scale bar = 1 mm. Average seed size (f) and average mean grey value (g) of different accession crosses. *n* = 3–4 pictures. Statistical differences were calculated via one‐way ANOVA with *post hoc* Tukey test (*P* < 0.05). Different letters represent different statistical groups. *Samplify* predictions and test statistics can be found in Supporting Information Tables S11–S18.

Finally, as *Samplify* was mainly developed to provide a quick and unbiased measure to distinguish mutants with different severities of the triploid block, we tested its sensitivity by predicting seed abortion in crosses of a genetic mutant that affects triploid block. Mutants in the MADS‐box transcription factor encoding gene *PHERES1* (*PHE1*) and its paralogue *PHE2* suppress the triploid block. The triploid block is restored when complementing the *phe1phe2* double mutant with *PHE1::PHE1‐GFP* (Batista *et al*., 2019). To test whether *Samplify* would identify these differences, we generated the same seed genotypes by pollinating Col‐0 maternal plants with pollen from either *omission of second division 1 (osd1)* mutants, *osd1phe2*, or *osd1phe1phe2*. Mutants in *OSD1* produce diploid gametes at high frequency (d'Erfurth *et al*., 2009) and induce a strong triploid block when used as pollen donor to pollinate diploid maternal plants (Kradolfer *et al*., 2013). Comparing *Samplify* predictions of F1 Col‐0 × Col‐0, F1 Col‐0 × *osd1*, F1 Col‐0 × *osd1phe2*, and F1 Col‐0 × *osd1phe1phe2*, we could observe the previously published phenotype of high proportions of fully collapsed seeds in F1 Col‐0 × *osd1* and F1 Col‐0 × *osd1phe2*, while strongly decreased numbers of fully collapsed seeds in F1 Col‐0 × *osd1phe1phe2* (Fig. 5a; Table S11). This significant difference in seed abortion in *osd1phe1phe2* compared with *osd1* and *osd1phe2* was also reflected in the seedling establishment, resulting in nearly 100% of seedlings established in Col‐0 and *osd1phe1phe2* pollinations, while only 25.9% and 38.6% in *osd1* or *osd1phe2* pollinations (Fig. 5b; Table S12). We noted that the seedling establishment rate in F1 Col‐0 × *osd1phe1phe2* was higher than anticipated from the *Samplify* prediction, likely attributed to the high rate of partially collapsed seeds in this genotype (which maintain a high germination potential (Fig. 3f) and lower confidence prediction of this particular seed category (Fig. 5b; Table S11)). Taken together, this shows that *Samplify* reliably predicts differences in seed abortion in different genotypes, making it a reliable tool to identify variation in seed abortion between different genotypes.

To further test the versatility of *Samplify*, we tested whether it would also reliably predict differences in triploid block between different *Arabidopsis thaliana* accessions. The *Arabidopsis* accession Landsberg *erecta* (L*er*) shows a weaker triploid block compared with the Col‐0 accession (Scott *et al*., 1998; Dilkes *et al*., 2008). We pollinated L*er*‐0 with pollen from autotretraploid L*er*‐0 plants (F1 L*er*‐0 × 4× L*er*‐0) and compared those seeds with their diploid controls (F1 L*er*‐0 × L*er*‐0). Moreover, we also included independent crosses of F1 Col‐0 × Col‐0 and F1 Col‐0 × 4× Col‐0, similar to the one shown in Fig. 3. Consistent with previously reported results, we found a lower triploid block in F1 L*er*‐0 × 4× L*er*‐0 with an average of 81.7% normal seeds, 9.3% partially collapsed seeds and 8.9% fully collapsed seeds, whereas the F1 Col‐0 × 4× Col‐0 had 6%, 15.6% and 78.4% of normal, partially collapsed and fully collapsed seeds, respectively (Fig. 5c; Table S13). Also, the average seedling establishment of F1 L*er*‐0 × 4× L*er*‐0 was significantly higher at 93.4% than that of F1 Col‐0 × 4× Col‐0 seeds showing only 30.2% of seedling establishment (Fig. 5d; Table S14). In conclusion, these data demonstrate that *Samplify* reliably predicts previously published differences of the triploid block between different accessions (Dilkes *et al*., 2008).

### *Samplify* can be used for different seed‐related phenotypes

Finally, we tested the application of *Samplify* beyond seed abortion prediction. *Samplify*'s accurate segmentation enables the collection of multiple individual parameters from a given seed picture, facilitating comparisons that do not require prior training of RF models. The L*er* accession is known to have smaller seeds than Col‐0 (Fig. 5e; Herridge *et al*., 2011, Ren *et al*., 2019). To test whether *Samplify*'s segmentation allows us to predict this difference, we extracted the seed area in pixels and converted it to mm^2^. Indeed, these data showed that F1 L*er*‐0 × L*er*‐0 were significantly smaller than F1 Col‐0 × Col‐0 (Fig. 5f; Table S15). Similarly, the different seed categories (normal, partially and fully collapsed) also showed characteristic size changes, resulting in smaller F1 L*er*‐0 × L*er*‐0 normal seeds, but larger F1 seeds in triploid block crosses as long as the seeds were not fully collapsed (Fig. S4A; Table S16). Moreover, we utilized the mean grey value from *Samplify*'s parameter list to test the color intensity of F1 seeds. Collapsed seeds are usually characterized by a dark brown color, which could be used as a fast assessment of the triploid block, without relying on a pretrained RF classifier. Indeed, F1 Col‐0 × 4× Col‐0 had a lower mean grey value than F1 Col‐0 × Col‐0 (Fig. 5g), reflective of the high number of collapsed seeds in the F1 Col‐0 × 4× Col‐0. By contrast, the mean grey value of F1 L*er*‐0 × L*er*‐0 was significantly increased compared with the other F1 populations (Fig. 5g; Table S17), consistent with L*er* having a lighter seed color than Col‐0 (Dilkes *et al*., 2008). This difference became apparent in the different seed categories, showing that especially partially and fully collapsed seeds are affected in color (Fig. S4B; Table S18). In conclusion, the results presented here show that *Samplify* can reliably identify accession‐specific differences in the triploid block, seed size and seed color. The hybrid segmentation of *Samplify* detects the vast majority of seeds in the picture and classifies them based on a pretrained RF model with high accuracy, confirmed by germination data as well as published phenotypes. Moreover, since segmentation in *Samplify* is independent of the trained RF model, key seed parameters, such as size or color (grey mean value), can be extracted directly from the parameter list. This enables rapid analysis of different seed populations, making *Samplify* a valuable tool even without a trained RF model, and extending its utility beyond the sole quantification of collapsed seeds.

## Discussion

Plant phenotyping and the (semi‐) automated characterization of different traits is a fast‐developing area. Quantification of seed phenotypes has been the focus of many research groups, leading to the development of numerous tools over the years (Tanabata *et al*., 2012; Whan *et al*., 2014; Wang *et al*., 2023; Wei *et al*., 2023). The majority of those tools use simple pictures as input, each usually containing multiple specimens to be described. As such, accurate detection and annotation of all single objects in a given picture is key. Thus, image segmentation is the first and arguably the most challenging part; it is affected by multiple factors, such as appropriate lighting, sufficient resolution, and often sufficient spacing between single objects, avoiding them from touching or overlapping. Moreover, the highly irregular shapes of collapsed seeds create additional challenges, resulting in frequent misannotation or the loss of substantial numbers.

Here, we present *Samplify* as a tool designed for processing *Arabidopsis* seed images using a hybrid segmentation approach. It combines classical image segmentation techniques with Meta's SAM. Using this zero‐shot learning algorithm, we were able to capture the majority of individual seeds in a single picture, even though they are touching or building larger clusters, which is difficult to identify with classical segmentation approaches. The combination of traditional segmentation together with SAM builds a robust, reliable, and fast tool for seed segmentation, requiring only colored images as input. It leads to high segmentation ratios, aligning almost perfectly with manually counted seeds. On average, *Samplify* processes a picture with *c*. 200 seeds in *c*. 100 s, resulting in 0.5 s per seed on our system (NVIDIA GeForce RTX 4070 GPU; see material and methods). The SAM by Meta has been widely applied in medical imaging tasks, such as tumor segmentation (Putz *et al*., 2025) and anatomical structure detection (Lei *et al*., 2025), but we are aware of only two studies applying SAM in plant phenotyping (Tang *et al*., 2024; Zhou *et al*., 2024). Here, we present one of the first applications of SAM for seed segmentation and its first application on *Arabidopsis* seeds.

The combination of balancing classical segmentation approaches and SAM algorithm has been shown to be effective in plant seed phenotyping with the release of GRABSEEDS (Tang *et al*., 2024). GRABSEEDS, however, was not developed and trained on small *Arabidopsis* seeds and not on seeds in different abortion stages, resulting in morphological irregularities interfering with accurate segmentation. When applied to representative *Arabidopsis* seed images from our collection, GRABSEEDS failed to correctly separate touching seeds. Unlike GRABSEEDS, *Samplify* does not require or recommend the manual separation of touching seeds, provided they do not lie on top of each other. Seed segmentation is case‐specifically balanced between Otsu's method and SAM. Regions with dense seed clusters are processed using SAM, whereas regions with well‐distributed individuals are segmented using Otsu's thresholding (Otsu, 1979).

*Samplify* was specifically designed for scoring seed abortion occurring in the triploid block, and we provide a trained RF model allowing the quantification of the different seed categories. The model has been trained on *c*. 13 000 seeds of different abortion stages in 99 pictures of Col‐0 F1 crosses, making it robust for scoring triploid block rates. Using *Samplify*, we were able to predict seed abortion with the same precision as manual counting, which was further validated by germination tests. Moreover, we could show that *Samplify* allows us to predict differences in the triploid block of previously identified suppressor mutants (Batista *et al*., 2019), making it a versatile screening tool for modifiers of the triploid block. Although the RF classifier was trained on Col‐0 data, it was able to predict that L*er* has a weaker triploid block (Dilkes *et al*., 2008). Even though the triploid block detection worked well using our Col‐0 based RF model and *Samplify*'s prediction is reliable across multiple biological samples and iterations, we encourage users to train their own models, as seed abortion prediction is model dependent, which in turn, is user and possibly hardware‐dependent (imaging).

Segmentation works similarly under different light conditions, yet predictions do vary likely due to changes in the color spectrum, which is, among other parameters, critical for training the RF classifier. *Samplify* corrects for different lighting conditions by applying a global background normalization; nonetheless, well‐exposed pictures resulted in the best prediction outcome.

At last, it is noteworthy that *Samplify*'s prediction works well for normal or fully collapsed seeds. However, partially collapsed seeds remain the most difficult category to distinguish, as these seeds arise from an ambiguous morphological range, which leads to inconsistencies between human annotations and algorithmic predictions. Due to that, seed populations with high ratios of partially collapsed seeds generally result in lower prediction confidence (Table S11), which becomes apparent in that it is triploid block suppressor mutants (Fig. 5a). Nonetheless, *Samplify* reduces the potential human bias, making the predictions more reliable and especially more comparable to each other compared with results from various human annotators.

*Samplify* was initially developed as a tool for fast and reliable annotation of seed abortion, yet its flexibility and strong segmentation capability make it useful in many other aspects. For example, we show that *Samplify* can be used for seed size or color comparisons, as exemplified by identifying differences in previously published seed size and color between different *Arabidopsis* accessions (Alonso‐Blanco *et al*., 1999; Dilkes *et al*., 2008; Herridge *et al*., 2011; Ren *et al*., 2019). To facilitate the exploration of different seed shape parameters, we included an extraction function to *Samplify*, enabling users to directly extract specific features used by *Samplify* side‐by‐side with annotation results.

In conclusion, *Samplify*, together with the pretrained RF model presented here, offers a fast, accessible, and reliable workflow for quantifying seed abortion across diverse *Arabidopsis* accessions and genetic mutants. By minimizing user‐dependent biases and leveraging a hybrid segmentation strategy that achieves near‐perfect accuracy while optimizing computational demands, *Samplify* provides a robust, versatile tool for plant developmental studies. Beyond seed abortion, its adaptable framework holds promise for broader applications in seed phenotyping and quantification.

## Competing interests

None declared.

## Author contributions

HB, CK and DW conceived and conceptualized the study. RLJM wrote and optimized the *Samplify* code and trained the models. AD installed and implemented the code base necessary for performing and hosting *Samplify*. HB performed the wet lab experiments. CK, HB, DW and RLJM analyzed the data. HB and CK wrote the manuscript. HB and RLJM contributed equally to this work and share first authorship. All authors read and commented on the manuscript.

## Disclaimer

The New Phytologist Foundation remains neutral with regard to jurisdictional claims in maps and in any institutional affiliations.

## Supporting information


**Fig. S1** Flowchart of the *Samplify* processing steps.
**Fig. S2** Relative feature importance of all features used by *Samplify* default RF model.
**Fig. S3** Effect of image resolution on *Samplify* predictions.
**Fig. S4** Size and mean grey value of differently annotated seed categories based on *Samplify* predictions.

**Table S1** A list of all computed seed shape parameters used by *Samplify* to predict seed abortion.
**Table S2** Accuracy of training and validation data from *Samplify* prediction using different training set sizes.
**Table S3** *Samplify* annotated seeds and test statistics complementary to Fig. 3(c).
**Table S4** Manually annotated seeds and test statistics, complementary to Fig. 3(d).
**Table S5** Total seeds counts per picture, counted manually and with *Samplify*.
**Table S6** Seedling establishment results, complementary to Fig. 3(f).
**Table S7** *Samplify* annotated seeds in categories, complementary to Fig. 4(a,b).
**Table S8** Reproducibility calculation results per seed population and abortion category, complementary to Fig. 4(b).
**Table S9** Relative *Samplify* prediction results for each category and seed population at different lighting condition, complementary to Fig. 4(d).
**Table S10** Relative *Samplify* prediction results for each category and seed population at different resolutions complementary to Fig. S3.
**Table S11** *Samplify* annotated seeds and test statistics for the triploid block suppressor mutants, complementary to Fig. 5(a).
**Table S12** Seedling establishment results for triploid block suppressor mutants, complementary to Fig. 5(b).
**Table S13** *Samplify* annotated seeds and test statistics for the triploid block in different accessions, complementary to Fig. 5(c).
**Table S14** Seedling establishment results for triploid block in different accessions, complementary to Fig. 5(d).
**Table S15** Average seed size values of all used accession pictures complementary to Fig. 5(f).
**Table S16** Average seed size values per seed category of all used accession pictures, complementary to Figs 5(f) and S4.
**Table S17** Mean Grey value of all used accession pictures, complementary to Fig. 5(g).
**Table S18** Mean Grey value per seed category of all used accession pictures, complementary to Figs 5(g) and S4.
Please note: Wiley is not responsible for the content or functionality of any Supporting Information supplied by the authors. Any queries (other than missing material) should be directed to the *New Phytologist* Central Office.

## Data Availability

*Samplify*, the RF model and detailed step‐by‐step user instructions are available on GitHub: https://github.com/Ronja‐Mueller/Samplify.git. The data that support the findings of this study are available in the Supporting Information of this article (Tables S1–18).
