# Supplementary material for: Samplify: a versatile tool for image‐based segmentation and annotation of seed abortion phenotypes
Source: New Phytol. 2026 Feb 15;250(3):1964–78. doi: 10.1111/nph.70979 (PMC13062712; doi:10.1111/nph.70979)
Supplement: Supplementary file 1 — Fig. S1 Flowchart of the Samplify processing steps. Fig. S2 Relative feature importance of all features used by Samplify default RF model. Fig. S3 Effect of image resolution on Samplify predictions. Fig. S4 Size and mean grey value of differently annotated seed categories based on Samplify predictions. [file NPH-250-1964-s001.docx]

## *New Phytologist* Supporting Information

Article title: *Samplify*: A versatile tool for image-based segmentation and annotation of seed abortion phenotypes

Authors: Heinrich Bente, Ronja Lea Jennifer Müller, Andreas Donath, Dirk Walther, Claudia Köhler

Article acceptance date: 20 January 2026

The following Supporting Information is available for this article:


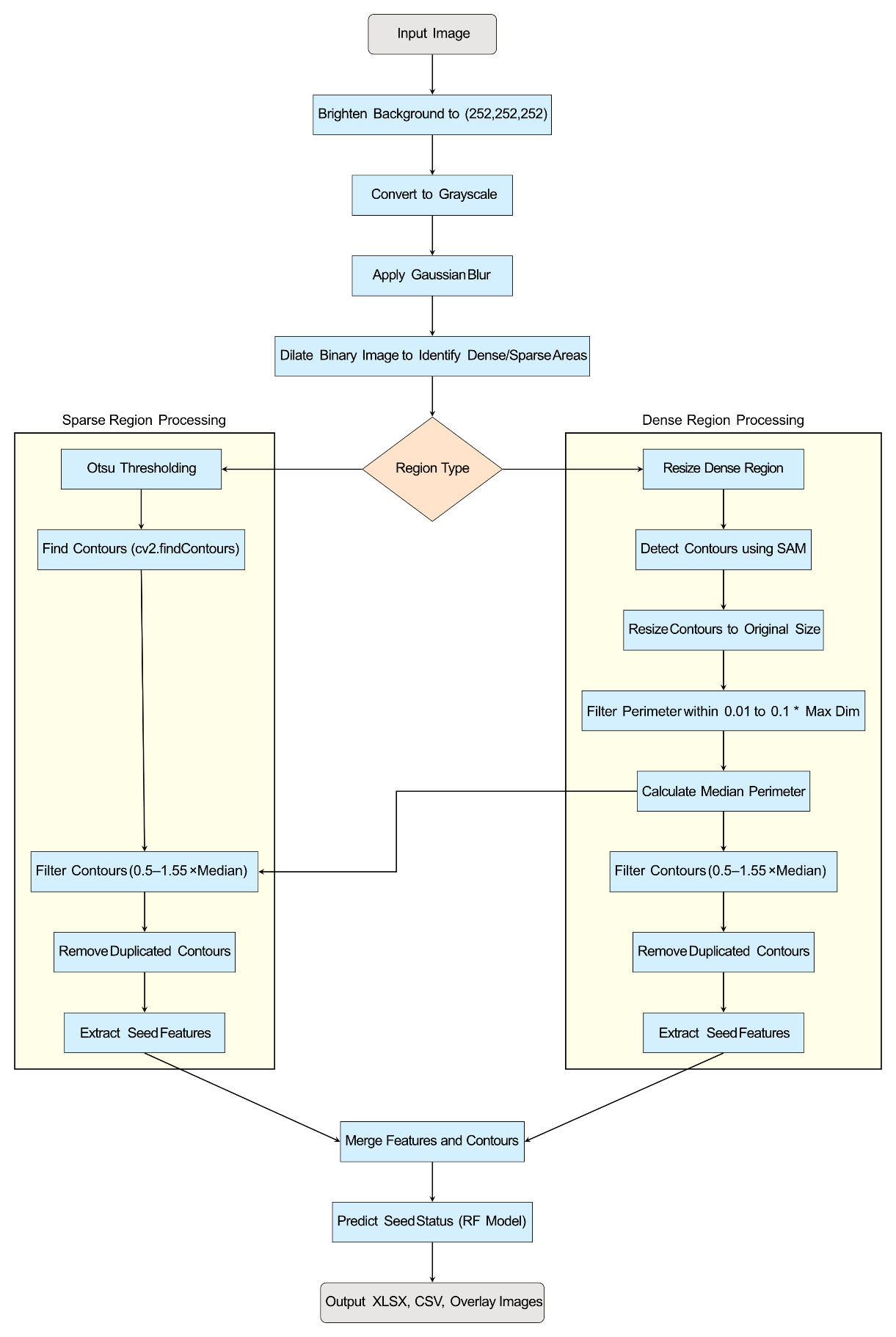


**Fig. S1** Flowchart of the *Samplify* processing steps.


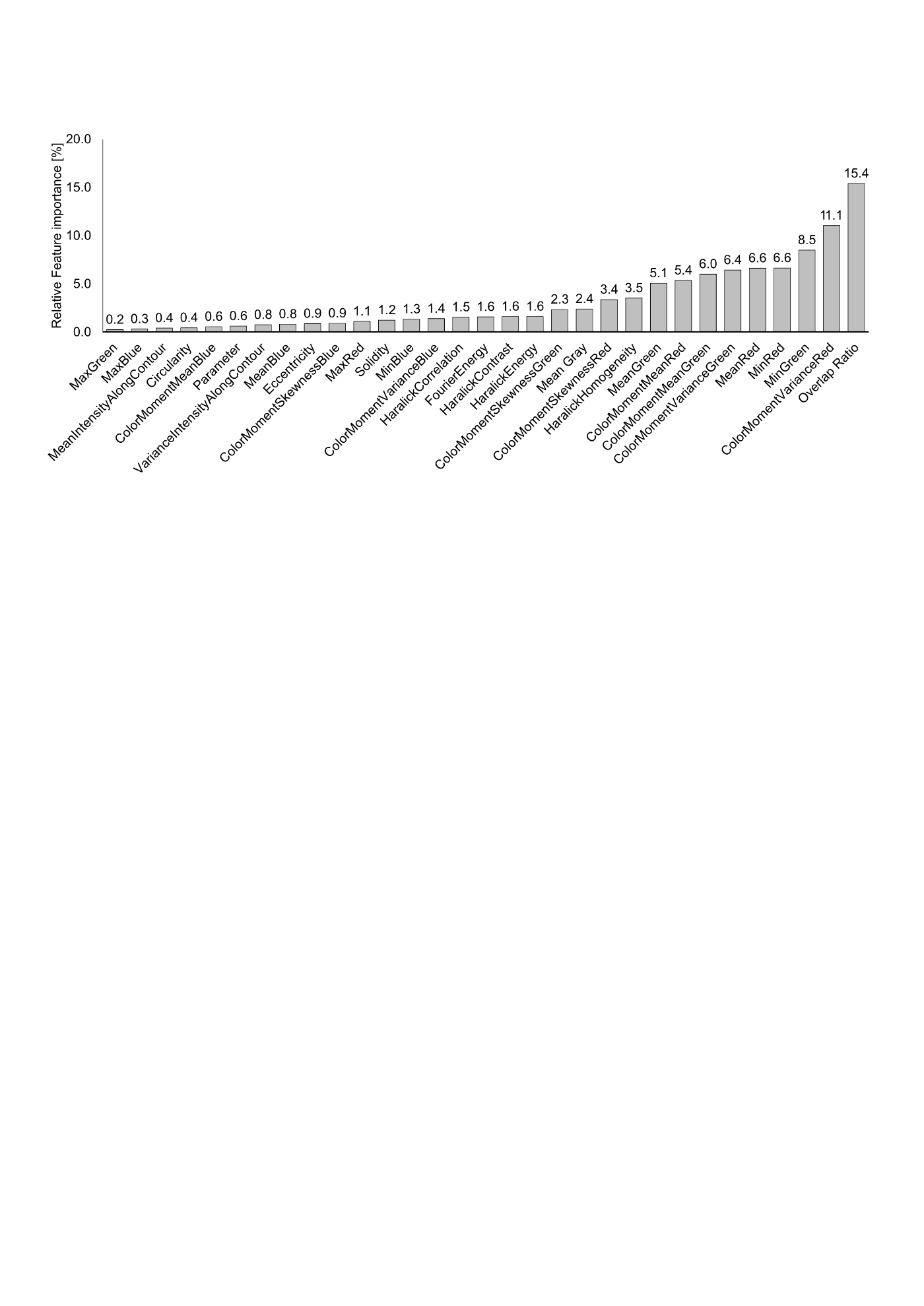


**Fig. S2** Relative feature importance of all features used by *Samplify* default RF model.


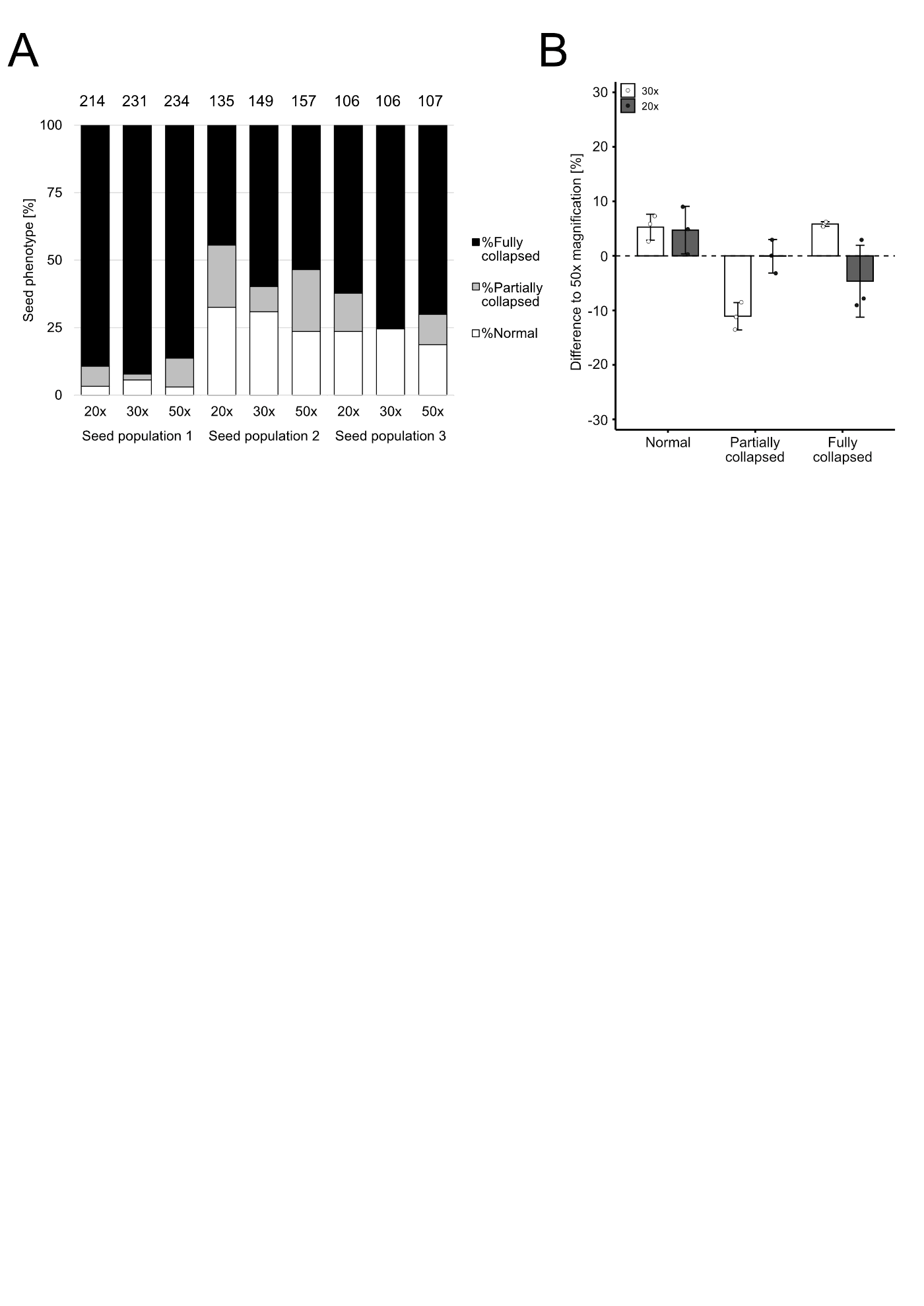


**Fig. S3** Effect of image resolution on *Samplify* predictions. A) Relative seed phenotype of three independent seed populations quantified by *Samplify*. The same seed population was imaged with 20x magnification (approx. 19 megapixel), 30x magnification (approx. 55 megapixel) and 50x magnification (approx. 130 megapixel). Number of detected seeds are indicated on top of bars. B) Change in relative seed phenotype predictions at different resolutions as shown in (A). Three seed populations were imaged at three different resolutions and the relative change in seed phenotype per category is shown, compared to the highest resolution. Error bars represent standard deviation. Relative values are denoted in Supplementary Table S10.


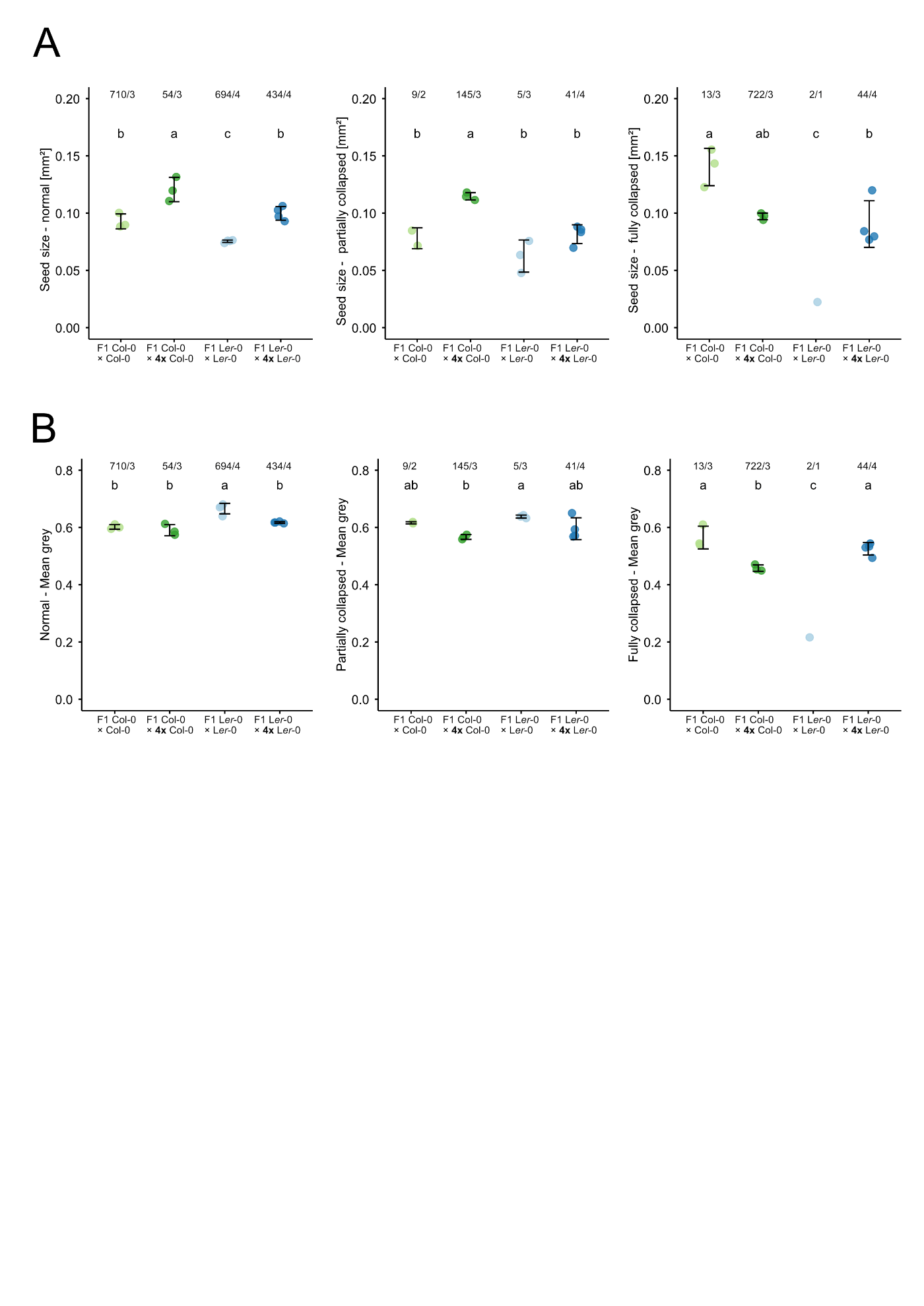


**Fig. S4** Size and mean grey value of differently annotated seed categories based on *Samplify* predictions. A) Average seed size in of seeds predicted to be normal (left panel), partially collapsed (middle panel) and fully collapsed (right panel) of different accession crosses. B) Average mean grey value in of seeds *predicted* to be normal (left panel), partially collapsed (middle panel) and fully collapsed (right panel) of different accession crosses. n = 3-4 pictures. Statistical differences were calculated via using one-way ANOVA with post-hoc Tukey test (p < 0.05). Different letters represent different statistical groups. Number on top represents the total number of seeds / number of images. *Samplify* predictions and test statistics can be found in Supplementary Tables S16 and S18.

**Table S1** A list of all computed seed shape parameters used by *Samplify* to predict seed abortion.

**Table S2** Accuracy of training and validation data from *Samplify* prediction using different training set sizes. Train_size: Number of seeds for each training set size; mean_train: Mean accuracy for training set prediction: std_train: Standard deviation of accuracy for training set prediction; ci95_train: 95% confidence interval for training set prediction; mean_val: Mean accuracy for validation set prediction: std_val: Standard deviation of accuracy for validation set prediction; ci95_train: 95% confidence interval for validation set prediction. Each trainings set size was randomly sampled from a ‘training pool’ with 10509 seeds, for 30 iterations. Different sized training sets were tested against an independent ‘validationpool’ with 2628 seeds.

**Table S3** *Samplify* annotated seeds and test statistics complementary to Fig. 3C. Model-estimated probabilities (± SE) that a seed from each genotype falls into the indicated phenotype category (Normal, partially collapsed, fully collapsed), based on a quasi-multinomial logistic regression model. Lower and upper confidence limits (lower.CL, upper.CL) represent 95% confidence intervals on the probability scale. Letters (group) indicate Tukey-adjusted significance groups within each phenotype category (estimated marginal means, emmeans). Pairwise genotype contrasts within each seed phenotype category from the quasi-multinomial model. Estimates are differences in model-estimated probabilities (± SE) with associated test statistics and Tukey-adjusted P values.

**Table S4** Manually annotated seeds and test statistics, complementary to Fig. 3C. Model-estimated probabilities (± SE) that a seed from each genotype falls into the indicated phenotype category (Normal, partially collapsed, fully collapsed), based on a quasi-multinomial logistic regression model. Lower and upper confidence limits (lower.CL, upper.CL) represent 95% confidence intervals on the probability scale. Letters (group) indicate Tukey-adjusted significance groups within each phenotype category (estimated marginal means, emmeans). Pairwise genotype contrasts within each seed phenotype category from the quasi-multinomial model. Estimates are differences in model-estimated probabilities (± SE) with associated test statistics and Tukey-adjusted P values.

**Table S5** Total seeds counts per picture, counted manually and with *Samplify*

**Table S6** Seedling establishment results, complementary to Fig. 3F. emmean: estimated marginal mean for each genotype; SE: standard error of the estimated marginal mean; df: degrees of freedom for the estimated marginal mean; asymp.LCL: asymptotic lower confidence limit for the estimated marginal mean. asymp.UCL: asymptotic upper confidence limit for the estimated marginal mean. group: statistical group label assigned. P-value: Tukey-adjusted P value.

**Table S7** *Samplify* annotated seeds in categories, complementary to Fig. 4A and 4B.

**Table S8** Reproducibility calculation results per seed population and abortion category, complementary to Fig. 4B. SD: Standard deviation; CV: Coefficient of Variation; (SD/mean); CV_percentage: CV*100. Reproducibly: 1-CV. NA indicates instances with less than 10 observations.

Table S9 Relative *Samplify* prediction results for each category and seed population at different lighting condition, complementary to Fig. 4D.

**Table S10** Relative *Samplify* prediction results for each category and seed population at different resolutions complementary to Supplementary Fig. S3. ‘Zoom’ depicts the used magnification resulting at a given scan area of 5x4 cm in approx. 19 megapixel (20x), 55 megapixel (30x) or 130 megapixel (50x).

**Table S11** *Samplify* annotated seeds and test statistics for the triploid block suppressor mutants, complementary to Fig. 5A. Model-estimated probabilities (± SE) that a seed from each genotype falls into the indicated phenotype category (Normal, Partially collapsed, Fully collapsed), based on a quasi-multinomial logistic regression model. Lower and upper confidence limits (lower.CL, upper.CL) represent 95% confidence intervals on the probability scale. Letters (group) indicate Tukey-adjusted significance groups within each phenotype category (estimated marginal means, emmeans).

**Table S12** Seedling establishment results for triploid block suppressor mutants, complementary to Fig. 5B. emmean: estimated marginal mean for each genotype; SE: standard error of the estimated marginal mean; df: degrees of freedom for the estimated marginal mean; asymp.LCL: asymptotic lower confidence limit for the estimated marginal mean. asymp.UCL: asymptotic upper confidence limit for the estimated marginal mean. group: statistical group label assigned.

**Table S13** *Samplify* annotated seeds and test statistics for the triploid block in different accessions, complementary to Fig. 5C. Model-estimated probabilities (± SE) that a seed from each genotype falls into the indicated phenotype category (Normal, partially collapsed, fully collapsed), based on a quasi-multinomial logistic regression model. Lower and upper confidence limits (lower.CL, upper.CL) represent 95% confidence intervals on the probability scale. Letters (group) indicate Tukey-adjusted significance groups within each phenotype category (estimated marginal means, emmeans).

**Table S14** Seedling establishment results for triploid block in different accessions, complementary to Fig. 5D. emmean: estimated marginal mean for each genotype; SE: standard error of the estimated marginal mean; df: degrees of freedom for the estimated marginal mean; asymp.LCL: asymptotic lower confidence limit for the estimated marginal mean. asymp.UCL: asymptotic upper confidence limit for the estimated marginal mean. group: statistical group label assigned.

**Table S15** Average seed size values in pixel and mm2 as well as ANOVA results of all used accession pictures, complementary to Fig. 5F.

**Table S16** Average seed size values per seed category in pixel and mm2 as well as ANOVA results of all used accession pictures, complementary to Fig. 5F and Supplementary Fig. S4.

**Table S17** Mean Grey value and ANOVA results of all used accession pictures, complementary to Fig. 5G.

**Table S18** Mean Grey value per seed category with ANOVA results of all used accession pictures, complementary to Fig. 5G and Supplementary Fig. S4.
